# Very low prevalence of IgE mediated wheat allergy and high levels of cross-sensitisation between grass and wheat in a UK birth cohort

**DOI:** 10.1186/s13601-016-0111-1

**Published:** 2016-06-22

**Authors:** Carina Venter, Kate Maslin, Syed Hasan Arshad, Veeresh Patil, Jane Grundy, Gillian Glasbey, Roger Twiselton, Taraneh Dean

**Affiliations:** The David Hide Asthma and Allergy Research Centre, St. Mary’s Hospital, Newport, Isle of Wight PO30 5TG UK; School of Health Sciences and Social Work, University of Portsmouth, James Watson West, 2 King Richard 1st Road, Portsmouth, PO1 2FR UK

**Keywords:** Wheat allergy, Cross-reactions, Cross-sensitization, Hay fever, Food allergy

## Abstract

**Background:**

Patients often report adverse reactions to wheat. Interpretation of sensitization to wheat pollen and flour with/without sensitization to grass pollen is a clinical problem.

**Aim:**

We set out to determine the prevalence of wheat allergy in a birth cohort (10/11 year olds) and investigate the usefulness of performing skin prick tests (SPT), specific IgE tests and component resolved diagnostics to wheat pollen and flour.

**Methods:**

The Food Allergy and Intolerance Research (FAIR) birth cohort included babies born on the Isle of Wight (UK) between September 2001–August 2002 (n = 969). Children were followed up at 1, 2, 3 and 10/11 years. 588 children had SPTs to wheat pollen and grass during the 10 year follow-up. 294 children underwent further SPT to wheat flour and 246 had specific IgE testing to wheat and grass.

**Results:**

Eight children underwent oral food challenges (OFC). We diagnosed 0.48 % (4/827; 95 % CI 0–1 %) children with wheat allergy based on OFC. 16.3 % (96/588) were sensitized to grass pollen, 13.4 % (79/588) to wheat pollen; 78 % (75/96) sensitized to both. Only one child was sensitized to wheat flour and wheat pollen, but not grass pollen. For specific IgE, 15.0 % (37/246) and 36.2 % (89/246) were sensitized to wheat and grass pollen, with 40.5 % (36/89) sensitized to both. Of the 37 children sensitized to wheat, 3 (8.1 %) were sensitized to omega 5 gliadin, 1 (2.7 %) to wheat lipid transfer protein and 1 to wheat gliadin.

**Conclusion:**

Clinicians should be aware of the high level of cross-sensitization when performing tests to wheat and grass pollen i.e. sensitisation to wheat specific IgE and wheat pollen SPT should be assessed in the presence of grass pollen SPT and/or specific IgE.

## Background

The reported prevalence of adverse reactions to wheat and gluten has escalated in the past few years reflected by the stark increase in sales of so-called “free-from” products. Clinicians are faced with wheat and gluten related reported problems on a regular basis, with very little evidence about the usefulness of skin prick tests (SPT) and specific IgE tests in the diagnostic work-up of wheat allergy. A number of studies have recently been published on component resolved diagnostics (CRD), indicating that these may be useful tests [1] in distinguishing those with true wheat allergy in an unselected cohort. The usefulness of these tests and ultimately using oral food challenges in wheat allergies is summarized in a recent review paper, highlighting the need for more studies [2]. In the few published studies utilising food challenges, a prevalence of wheat allergy of 0.1–0.6 % has been reported in Europe [3–6], with the highest rate of 0.6 % in Italy [7]. Similar rates have been reported in the USA, by Bock et al. [8] and Vierk et al. [9].

Wheat contains cross-reacting proteins to both other cereals and pollens. It is composed of four classes of proteins; albumins, globulins, gliadins and glutenins, which together are known as prolamins or gluten. Of these, there are two major proteins considered to lead to adverse reactions, the lipid transfer protein (LTP) and the omega-5 gliadins (both considered to be prolamins or seed storage proteins) [10]. Wheat is likely to cross-react with grass, but this does not indicate the likelihood of an allergic reaction to consumption of wheat [11–13]. Wheat also contains a number of proteins that may cross-react with rye and barley.

Three types of IgE mediated wheat allergies have been well described in the past. The first is bakers’ asthma; resulting from inhaling flour or wheat dust. The main wheat proteins implicated in this are the a-amylase/LTPs [14]. The second form of wheat allergy is wheat-dependent exercise-induced anaphylaxis (WDEIA), mainly characterized by sensitization to the omega 5-gliadins. A third type of wheat allergy has also been described, often characterized by sensitization to the lipid transfer protein in wheat and the reactions can range from mild to more severe symptoms [15].

To our knowledge, the prevalence of wheat and grass cross-reactions and their clinical implications has only been studied in one unselected birth cohort. The German Multi-Centre Allergy study reported that sensitization to wheat was mostly secondary to pollen sensitisation at school age [16]. In our study, we set out to determine the prevalence of wheat allergy and intolerance in a birth cohort of 10–11 year old children and investigate the particular usefulness of performing SPT to wheat pollen, wheat flour, specific IgE to wheat and wheat CRD as well as cross-sensitization to grass pollens in diagnosing wheat allergy.

## Methods

In brief, a whole population birth cohort was established on the Isle of Wight (UK) [6]. All pregnant mothers with an estimated delivery date of September 2001 to August 2002 were approached at antenatal clinics and infants were followed up prospectively. Children were followed up at 1, 2, 3 and 10/11 years. Children were clinically examined and had SPT using ALK Abello to a standard battery of food allergens (milk, egg, *wheat pollen*, peanut, fish), aero-allergens (house dust mite *Dermatophagoides pteronyssinus* (HDM), cat and grass) and other allergens if identified by history, as described previously [6].

Reported symptoms of hay fever and asthma using the ISAAC [17] questions as well as reported symptoms to wheat and other foods were recorded, and those with a convincing history of adverse reactions to wheat were invited for food challenges. Food challenges were performed using the PRACTALL [18] guidelines for IgE mediated food allergy. To diagnose non-IgE mediated food allergy or intolerance, an age appropriate daily amount of wheat was given to the individual. Food challenges were performed blinded where possible.

Children were invited back for a further SPT to *wheat flour* as initial wheat SPT were performed with pollen. They were also asked to undergo a blood test using the fx5 Immunocap (milk, egg, wheat, cod, soy and peanut) and the Phadia aero-allergen screen test (a balanced mixture including grasses, trees, weeds, cat, dog, mites and moulds). If these tests were positive, specific IgE tests to the individual allergens were performed. Specific IgE tests to whole food protein and recombinant selected protein were classified as positive at 0.35 kUA/l. CRD were performed to omega-5 gliadin (Tri a 19), gliadin (alpha, gamma, beta and omega gliadin) and the wheat LTP (Tri a 14). For grass we measured grass profilin (Phlp 12) and three further grass components (Phlp 1, Phlp 5b and Phlp 7).

### Ethics, consent and permission

Ethical approval for the study was obtained from the NRES South Central—Southampton B Research Ethics Committee (REF 10/H0504/11). All parents consented and children provided assent for the study.

### Statistical analysis

Data was double entered and verified and statistical analysis was conducted using SPSS version 21 (IBM SPSS Statistics, IBM Corporation, Armonk, NY). Descriptive statistics and 2 × 2 tests were used to describe the sample.

## Results

At the age of 10 years, 827/969 (85 %) of the original cohort was followed up. SPT was performed in 588 children (71 % of those with questionnaire data) to a panel that included wheat pollen. 294 children attended for a second visit to have SPT to wheat flour and 246 (41.8 %) children consented to have a blood test. Follow up and assessment data is summarized in Table 1.

**Table 1:** Sensitization to wheat and grass allergens in an unselected cohort

Characteristics	N (%)	
Reported a problem to wheat	17/827 (20.6)	
Avoided wheat	9/827 (10.9)	
Positive OFC	4/827 (4.8)	
*SPT Data*		
Sensitized to grass (SPT)	96/588 (16.3)	
Sensitized to wheat pollen (SPT)	79/588 (13.4)	
Sensitized to wheat flour	1/588 (0.17)	
Sensitized to wheat pollen and grass pollen (SPT)	75/96 (78)	
Sensitization to wheat flour and grass pollen (SPT)	0/75 (0)	
Sensitized to wheat flour and wheat pollen (SPT)	1/66 (1.5)	

Specific IgE	Using 0.35 kUA/l as a cut off	Using 0.01 kUA/l as a cut off
Specific IgE positive to wheat	37/246 (15.0) rTria19 (omega 5 gliadin): 3/37 who were sensitised to wheat (8.1) Wheat LTP: 1/37 who were sensitised to wheat (2.7) Gliadin: 1/37 who were sensitised to wheat (2.7)	59/246 (24.0)
Specific IgE positive to grass	Timothy 89/246 (36.2) Phlp1 79/89 who were sensitised to grass (88.7) Phlp7 4/89 who were sensitised to grass (4.5) Phlp p12 (profilin) 14/89 who were sensitised to grass (15.7) Phlp 5b 47/89 who were sensitised to grass (52.8)	98/246 (39.8)
Sensitization to grass and wheat (specific IgE)	36/89 (40.5)	56/98 (57.2)
*SPT versus Specific IgE*		
Sensitized to wheat flour (SPT) and wheat specific IgE	1/36 (2.8)	
Sensitized to wheat pollen (SPT) and wheat (specific IgE)	31/36 (86.1)	
Sensitised to grass SPT and grass specific IgE	59/88 (67.0)	

As only 41.8 % of those who consented to SPT attended another appointment for a blood test, we compared those who consented versus those who did not. There was no difference in reported family history between the two groups. However, looking at any allergen sensitization over 10 years, 39.4 % of those who had a blood test had a history of sensitization, compared to 18.9 % of those who did not have a blood test (p < 0.05). We therefore feel that our data regarding wheat allergy and reported hay fever, asthma and SPT is a true reflection of an unselected population, but our data on specific IgE may be reflective of a higher risk population.

### Sensitization rates

#### Skin prick tests

16.3 % (96/588) children were sensitized to grass pollen and 13.4 % (79/588) to wheat pollen with 78 % (75/96) sensitized to both. Only one child was sensitized to wheat flour; also sensitized to wheat pollen but not to grass pollen.

#### Specific IgE tests

Using 0.35 kUA/l as a cut-off point, 15.0 % (37/246) and 36.2 % (89/246) were sensitized to wheat and grass pollen with 40.5 % (36/89) sensitized to both.

Using 0.1 kUA/l as a cut-off point, 24.0 % (59/246) and 39.8 % (98/246) were sensitized to wheat and grass pollen with 57.2 % (56/98) sensitized to both.

86.1 % of those sensitized to wheat pollen (SPT) were also sensitized to wheat specific IgE tests and 67.0 % sensitized to grass (SPT) were also sensitized to grass specific IgE tests.

**Fig. 1:**
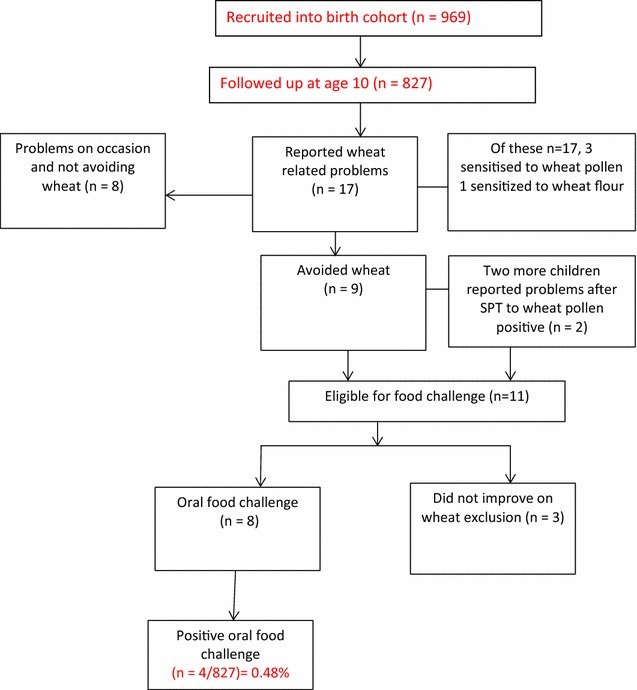
Reported wheat related problems and diagnosed wheat allergy

Figure 1 shows details of reported wheat related problems. Oral food challenge (OFC) was indicated in 8 children. Four were diagnosed with wheat allergy; one child with immediate symptoms. These children are further described in Table 2. The prevalence of wheat allergy based on oral food challenge (OFC) was 0.48 % (4/827; 95 % CI 0–1 %) and 0.12 % for IgE mediated wheat allergy (1/827; −0.12–0.36 %).

**Table 2:** Characteristics of children diagnosed with wheat allergy (n = 4)

Child	Characteristics
1. Wheat intolerance	Positive SPT wheat pollen Positive SPT grass No SPT to wheat flour No bloods Symptoms: GI symptoms
2. Wheat intolerance	Negative SPT wheat pollen and wheat flour Positive SPT grass Positive specific IgE Timothy grass Positive phlp1 Positive Phlp5b Positive Wheat specific IgE Negative to all wheat CRD Symptoms: GI symptoms
3. Wheat intolerance	Positive OFC no SPT or blood Symptoms: GI symptoms
4. IgE mediated wheat allergy	*Positive SPT wheat pollen* *Positive SPT to wheat flour* Negative SPT grass Positive food allergen screen Positive aero-allergen screen Positive Timothy grass specific IgE Positive Phlp1 Positive Phlp5b Positive Wheat specific IgE Positive Tria19 (omega 5 gliadin) Positive tri a 14 (LTP) Positive f98 (gliadins) *Symptoms: wheeze, rhinorrhoea, angioedema and vomiting*

### Reported symptoms of hay fever and asthma

**Fig. 2:**
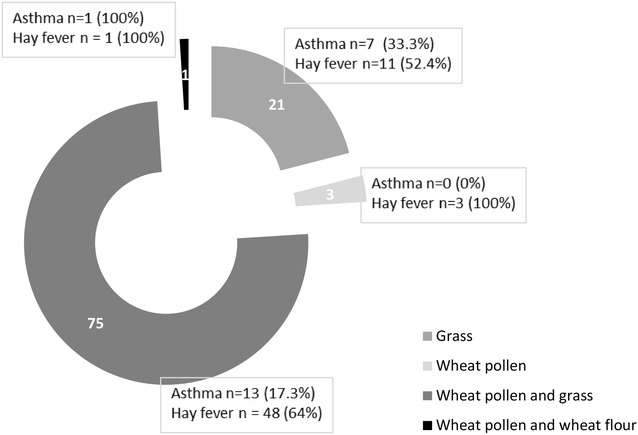
Sensitization patterns using skin prick tests (n = 100)

**Fig. 3:**
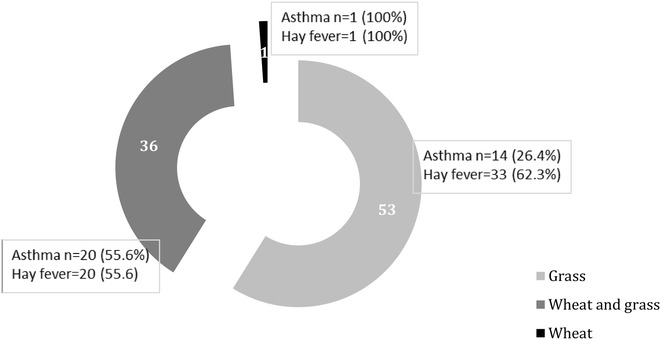
Sensitization patterns using specific IgE tests (n = 90)

Over the course of the first 10 years of life, 101 (12.2 %; 95 % CI 9.97–14.43 %) and 233 (28.2 %; 95 % CI 25.13–31.27 %) children reported to have suffered from asthma and hay fever respectively. Using SPT, of the 100 who were sensitized to grass using, wheat pollen or wheat flour, 75 were sensitized to both wheat pollen and grass. Using specific IgE tests, of the 90 who were sensitized to grass or wheat,  36 were sensitized to both wheat pollen and grass. Figures 2 and 3 show the sensitization patterns of these participants using SPT and specific IgE.

### Component testing

Of 37 children sensitized to wheat, 3 (8.1 %) were sensitized to omega 5 gliadin, 1 child (2.7 %) was sensitized to the wheat LTP and 1 child to the wheat gliadin.

In terms of the 89 children sensitized to timothy grass, 79 children (88.8 %) were sensitized to Phlp1, 4 (4.5 %) to Phlp7, 14 (15.7) to Phlp 12 (profilin) and 47 (52.8 %) to Phlp 5b.

## Discussion

In this study, we found a reported rate of wheat related symptoms of 2.1 % which is similar to that reported by Greenhawt et al. [19] in a similar age group from the USA and slightly higher than the EAACI systematic review of 1.5 % [20], however the one child with a wheat flour positive SPT result did not show a positive SPT to grass. Our diagnosed wheat allergy rate of 0.48 % is also similar to what we have previously reported on the Isle of Wight [5] and higher than the 0.3 % described by EAACI [20]. In our cohort we have only had one child (the same child) with IgE mediated wheat allergy at 1, 2, 3, and 10 years of age.

We found a high rate (78 %) of cross sensitization between grass SPT and wheat pollen SPT. Focusing on the specific IgE testing, we also found high levels of cross sensitization between wheat and grass with 40.5 % sensitized to wheat and grass. Taking into account that 37 children were sensitized to wheat, almost all (n = 36) of the wheat-sensitized children by means of specific IgE testing were also sensitized to grass (full details of this child’s sensitization status is described in Table 2).

Jones et al. reported in 1995 [12] that 72 % of children referred to a tertiary referral centre in the USA were sensitised to both wheat and grass using SPT (but a different reagent to that used in our study; Greer Laboratories). This is very similar to our data from an unselected cohort of children. However in their study 82 children were sensitized to wheat and 26 (32 %) had a positive OFC, whereas we could only diagnose IgE mediated wheat allergy in one child. Another child with a positive SPT to wheat pollen had a positive challenge, but the symptoms were delayed and gastro-intestinal only in nature, not suggestive of true IgE mediated wheat allergy. This discrepancy could be due to the fact that they were reporting on a secondary/tertiary care allergy population while we were reporting data on a birth cohort and we have indicated that our data on SPT should be reflective of a general population.

None of the grass-sensitized individuals were sensitized to the individual wheat components (gliadins, omega-5 gliadin and wheat LTP). Our numbers are very small which makes it difficult to compare with other studies, but as in the study with Makela [1], we found wheat CRD useful in the diagnosis of wheat allergy. The one child sensitized to the wheat LTP and gliadin was clinically reactive and one of three children sensitized to omega-5 gliadin was reactive. As this is a cohort of children we did not have any reported baker’s asthma, but the one child with IgE mediated wheat allergy does suffer from wheeze upon inhaling wheat, and is sensitized to the gliadins in wheat as in the study by Nam et al. [14] and Baar et al. [21.].

A strength of our study is that that we were able to describe wheat and grass cross-sensitization in a population based cohort and were able to look at the clinical relevance of the tests in terms of asthma, hay fever and wheat allergy supporting current literature [2, 22]. The study did however have three limitations: only 30 % of those that provided questionnaire information at 10 years consented to a blood test. We were only able to diagnose four children with a wheat allergy/intolerance of which only one had IgE mediated wheat allergy. It would have been helpful to look at other grains in addition to wheat in order to increase our understanding of clinical cross-reactivity between different grains.

Therefore in conclusion, we suggest that based on our limited data, SPT to wheat flour and CRD to wheat gliadins and LTP may be more useful in the diagnosis of wheat allergy than SPT to wheat pollens and wheat specific IgE. Based on only a small number of cases, the only tests that seem to show clinical relevance for the diagnosis of IgE mediated wheat allergy were SPT to wheat flour, CRD to wheat LTP and the gliadins. Although this data is from a birth cohort, rather than a clinical setting, it was shown that performing SPT to grass pollen and specific IgE merely indicates clinically irrelevant cross-sensitization. Care should be taken with interpretation of results when dealing with sensitization to wheat allergens.

## Abbreviations

SPT: skin prick test
LTP: lipid transfer protein
CRD: component resolved diagnostics
OFC: oral food challenge
